# Prognostic value of metabolic parameters on ^18^F-fluorodeoxyglucose positron tomography/computed tomography in classical rectal adenocarcinoma

**DOI:** 10.1038/s41598-021-92118-x

**Published:** 2021-06-21

**Authors:** Byung Wook Choi, Sungmin Kang, Sung Uk Bae, Woon Kyung Jeong, Seong Kyu Baek, Bong-Il Song, Kyoung Sook Won, Hae Won Kim

**Affiliations:** 1 Department of Nuclear Medicine, Daegu Catholic University Medical Center, Daegu Catholic University School of Medicine, Daegu, Republic of Korea; 2 Division of Colorectal Surgery, Department of Surgery, Keimyung University Dongsan Hospital, Keimyung University School of Medicine, Daegu, Republic of Korea; 3 Department of Nuclear Medicine, Keimyung University Dongsan Hospital, Keimyung University School of Medicine, 1035, Dalgubeol-daero, Dalseo-gu, Daegu, Republic of Korea

**Keywords:** Biomarkers, Gastroenterology, Molecular medicine, Oncology, Risk factors

## Abstract

We aimed to investigate the prognostic value of the metabolic parameters of ^18^F-fluorodeoxyglucose positron emission tomography/computed tomography (FDG PET/CT) in classical rectal adenocarcinoma (CRAC). We retrospectively reviewed 149 patients with CRAC who underwent preoperative ^18^F-FDG PET/CT at initial diagnosis followed by curative surgical resection. ^18^F-FDG PET/CT metabolic parameters including maximum standardized uptake value (SUVmax), metabolic tumour volume (MTV), and total lesion glycolysis (TLG) for disease-free survival (DFS) and overall survival (OS) were evaluated for prognostic significance by univariate and multivariate analyses, along with conventional risk factors including pathologic T (pT) stage, lymph node (LN) metastasis, lymphovascular invasion (LVI), perineural invasion (PNI), and preoperative carcinoembryonic antigen (CEA) level. On univariate analysis, high pT stage, positive LN metastasis, LVI, PNI, MTV, and TLG were significant prognostic factors affecting DFS (all *P* < 0.05), while CEA level, high pT stage, positive LN metastasis, LVI, PNI, MTV, and TLG affected OS (all *P* < 0.05). On multivariate analysis, positive LN metastasis, LVI, MTV, and TLG were independent prognostic factors affecting DFS (all *P* < 0.05), while CEA level, positive LN metastasis, and MTV affected OS (all *P* < 0.05). Thus, the volume-based metabolic parameters from preoperative ^18^F-FDG PET/CT scans are independent prognostic factors in patients with CRAC.

## Introduction

Globally, colorectal cancer is the third most commonly diagnosed cancer with an estimated 1.8 million new cases and is also the second leading cause of cancer-related deaths in 2018 according to the World Health Organization GLOBOCAN database^1^. Among the overall cases of colorectal cancer, almost 704,376 new cases were diagnosed as rectal cancer, with an estimated 310,394 cancer-related deaths. Multiple factors, including dietary patterns, cancer screening programs, and accessibility to optimal therapies have been affecting the change of trends in rectal cancer incidence and mortality^2^. Also, it is well known that the prognosis differs according to the histological types of rectal cancer^3–9^, and the adoption of a new treatment and proper management for rectal cancer patients could be one of the key players in the reduction of mortality. In this context, accurate and efficient prognostic biomarkers are needed for better clinical decision making in risk stratification, personalized treatment, care strategy, and prognostication in patients with rectal cancer.

A stepwise approach with multiple imaging modalities, such as ultrasonography, computed tomography (CT) scanning, magnetic resonance imaging (MRI), and fluorine-18-fluorodeoxyglucose (^18^F-FDG) positron emission tomography/CT (PET/CT) has been recommended for patients with rectal cancer in diagnosis, staging, treatment decision, response evaluation, and detection of recurrence^10,11^. Among these, ^18^F-FDG PET/CT, which reflects glucose utilization of target tissues, not only allows the optimal choice of therapeutic management but also assesses prognosis^12–16^. Previous studies with several kinds of malignancies have reported the clinical usefulness of using metabolic parameters of ^18^F-FDG PET/CT, such as maximum standardized uptake value (SUVmax), metabolic tumour volume (MTV), and total lesion glycolysis (TLG), for prediction of prognosis^17–20^. However, there are only a few studies focusing on the prognostic value of metabolic parameters in patients with rectal cancer^21–25^. Furthermore, these studies are limited in proving the prognostic value of metabolic parameters, because they used only the SUVmax to predict prognosis^26,27^. In addition, a previous study showed that classical rectal adenocarcinoma (CRAC), the most common histological subtype of colorectal cancer, had different clinical features and prognosis than other histological subtypes such as mucinous adenocarcinoma or signet-ring cell carcinoma^28^. However, there have been no studies focusing on the prognostic value of metabolic parameters on ^18^F-FDG PET/CT in CRAC alone. Thus, further studies with consideration of various kinds of metabolic parameters are needed in patients with CRAC.

The purpose of this study was to evaluate the prognostic value of metabolic parameters measured by ^18^F-FDG PET/CT in patients with CRAC.

## Results

### Patient characteristics

**Figure 1:**
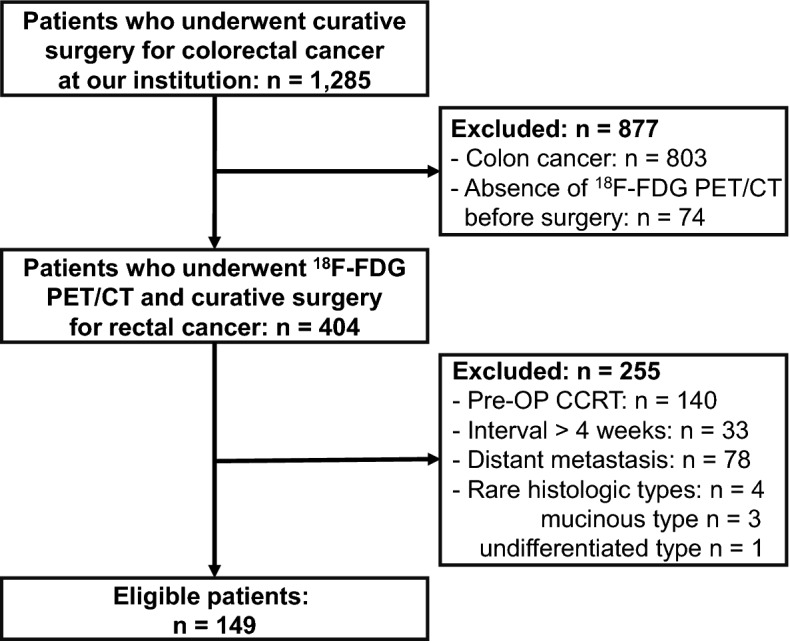
Flow chart of patient selection.

Of 404 patients who underwent preoperative ^18^F-FDG PET/CT and followed curative surgery for rectal cancer, 255 patients were excluded from this study according to the exclusion criteria (Fig. 1). A total of 149 patients with CRAC were included in this study; their demographic and clinicopathologic characteristics are shown in Table 1. Tumour recurrence was observed in 23 patients; the median follow-up time to recurrence was 13.1 months (mean ± standard deviation [SD]: 17.4 ± 14.1 months, range: 5.2–59.8 months). Cancer-related death occurred in 11 (7.4%) of these 23 patients; the median follow-up time to cancer-related death was 30.2 months (mean ± SD: 32.7 ± 17.8 months, range 13.2–74.1 months).

**Table 1:** Patient characteristics.

Characteristics	Value
Number of patients	149
Sex (M/F)	81/68
Age	66.3 ± 9.9
Follow-up time (months)	61.0 ± 23.6
Preoperative serum CEA level (ng/mL)	5.8 ± 22.9
T stage*	
Tis/1	25 (16.8%)
T2	40 (26.8%)
T3	69 (46.3%)
T4	15 (10.1%)
N stage*	
0	91 (61.1%)
1	33 (22.1%)
2	25 (16.8%)
Stage, pathologic*	
I	55 (36.9%)
II	37 (24.8%)
III	57 (38.3%)
**Histologic grade, *n***	
Well-differentiated	5 (3.4%)
Moderately differentiated	142 (95.3%)
Poorly differentiated	2 (1.3%)
**Lymphovascular invasion**	
Negative	78 (52.3%)
Positive	71 (47.7%)
**Perineural invasion**	
Negative	119 (79.9%)
Positive	30 (20.1%)
**Adjuvant therapy**	
No	75 (50.3%)
Yes	74 (49.7%)
**Recurrence**	
No	126 (84.6%)
Yes	23 (15.4%)
**Cancer-related death**	
No	138 (92.6%)
Yes	11 (7.4%)

Values are presented as means ± standard deviation or numbers of patients.

*CEA* carcinoembryonic antigen.

*According to the American Joint Committee on Cancer (AJCC) 7th edition.

### Prognostic factors

**Figure 2:**
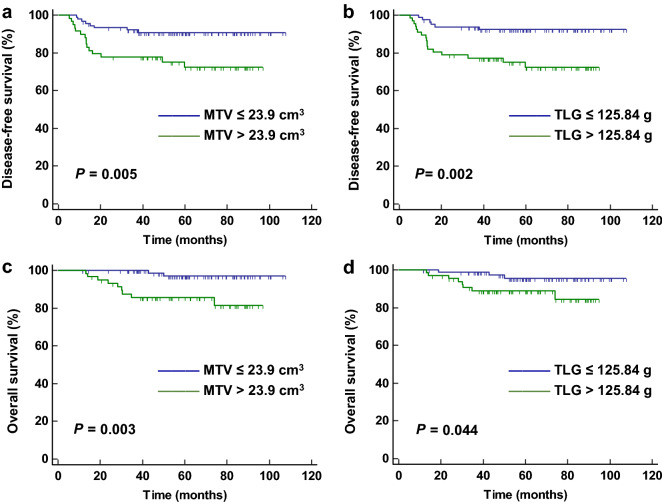
Kaplan–Meier curves for disease-free survival (DFS) and overall survival (OS) according to metabolic tumour volume (MTV) and total lesion glycolysis (TLG). Kaplan–Meier curves for DFS according to MTV (**a**) and TLG (**b**), and Kaplan–Meier curves for OS according to MTV (**c**) and TLG (**d**) in patients with classical rectal adenocarcinoma (n = 149).

In the univariate analyses of disease-free survival (DFS), pathologic T (pT) stage, positive lymph node (LN) metastasis, lymphovascular invasion (LVI), perineural invasion (PNI), MTV (Fig. 2a), and TLG (Fig. 2b) were significant prognostic factors. In the univariate analyses of overall survival (OS), preoperative serum carcinoembryonic antigen (CEA) level, pT stage, positive LN metastasis, LVI, PNI, MTV (Fig. 2c), and TLG (Fig. 2d) were significant prognostic factors. The SUVmax was not a significant prognostic factor for both DFS and OS. These results are summarized in Table 2.

**Table 2:** Mean survival time and univariate analysis of disease-free survival and overall survival.

Variables	DFS		OS	
	Mean, months	*P* value	Mean, months	*P* value
**Age at diagnosis, years**		0.504		0.389
≤ 50	61.7		72.2	
> 50	56.5		60.3	
**Sex**		0.780		0.979
Man	56.4		61.5	
Woman	57.1		60.4	
**Preoperative CEA level, ng/mL**		0.227		0.021*
≤ 5	58.9		63.0	
> 5	44.9		49.6	
**Pathologic T stage**		0.002*		< 0.001*
T1/2	61.2		62.8	
T3/4	53.3		59.5	
**Lymph node metastasis**		< 0.001*		0.004*
Negative	59.1		61.4	
Positive	53.1		60.3	
**Lymphovascular invasion**		0.001*		0.018*
Negative	60.5		62.4	
Positive	52.6		59.4	
**Perineural invasion**		0.001*		0.021*
Negative	59.4		62.5	
Positive	16.1		55.0	
**SUVmax**		0.453		0.333
≤ 14.2	56.7		58.8	
> 14.2	56.8		63.2	
**MTV, cm**^**3**^		0.005*		0.003*
≤ 23.9	60.0		63.0	
> 23.9	51.7		57.9	
**TLG, g**		0.002*		0.044*
≤ 125.84	62.1		64.1	
> 125.84	50.2		57.1	

*CEA* carcinoembryonic antigen, *DFS* disease-free survival, *MTV* metabolic tumour volume, *OS* overall survival, *SUVmax* maximum standardized uptake value, *TLG* total lesion glycolysis.

The Kaplan-Meier method was used, and statistical significance was determined using the log-rank test.

*Statistically significant.

As there was a significant correlation between MTV and TLG (r = 0.966, *P* < 0.001), MTV and TLG were assessed separately for multivariate analysis as model 1 (with MTV) and model 2 (with TLG). On the multivariate analyses, positive LN metastasis, LVI, MTV, and TLG were statistically significant independent prognostic factors for DFS (Table 3). Likewise, the multivariate analysis for DFS, MTV, and TLG was assessed separately from the multivariate analysis for OS. On the multivariate analyses, preoperative CEA level, positive LN metastasis, and MTV were statistically significant independent prognostic factors for OS (Table 4).

**Table 3:** Multivariate analysis of prognostic factors for disease-free survival.

Variables	Multivariate model 1^§^		Multivariate model 2^§^	
	HR (95% CI)	*P* value	HR (95% CI)	*P* value
Pathologic T stage (T1/2 vs. T3/4)		0.352		0.289
Lymph node metastasis (negative vs. positive)	3.02 (1.12–8.13)	0.029*	4.31 (1.69–11.02)	0.002*
Lymphovascular invasion (negative vs. positive)	2.86 (1.01–8.13)	0.049*		0.050
Perineural invasion (negative vs. positive)		0.294		0.170
MTV (≤ 23.9 vs. > 23.9)	2.47 (1.03–5.90)	0.042*		
TLG (≤ 125.84 vs. > 125.84)			3.21 (1.25–8.20)	0.015*

*CI* confidence interval, *HR* hazard ratio, *MTV* metabolic tumour volume, *TLG* total lesion glycolysis.

^§^MTV (model 1) and TLG (model 2) were separately assessed in a stepwise multivariate Cox proportional hazards model.

*Statistically significant.

**Table 4:** Multivariate analysis of prognostic factors for overall survival.

Variables	Multivariate model 1^§^		Multivariate model 2^§^	
	HR (95% CI)	*P* value	HR (95% CI)	*P* value
Preoperative CEA level (≤ 5 vs. > 5)		0.070	3.60 (1.04–12.46)	0.043*
Pathologic T stage (T1/2 vs. T3/4)		0.522		0.097
Lymph node metastasis (negative vs. positive)	5.62 (1.20–26.31)	0.029*	7.16 (1.55–33.20)	0.012*
Lymphovascular invasion (negative vs. positive)		0.161		0.181
Perineural invasion (negative vs. positive)		0.432		0.232
MTV (≤ 23.9 vs. > 23.9)	5.65 (1.20–26.51)	0.028*		
TLG (≤ 125.84 vs. > 125.84)				0.174

*CEA* carcinoembryonic antigen, *CI* confidence, *HR* hazard ratio, interval, *MTV* metabolic tumour volume, *TLG* total lesion glycolysis.

^§^MTV (model 1) and TLG (model 2) were separately assessed in a stepwise multivariate Cox proportional hazards model.

*Statistically significant.

**Figure 3:**
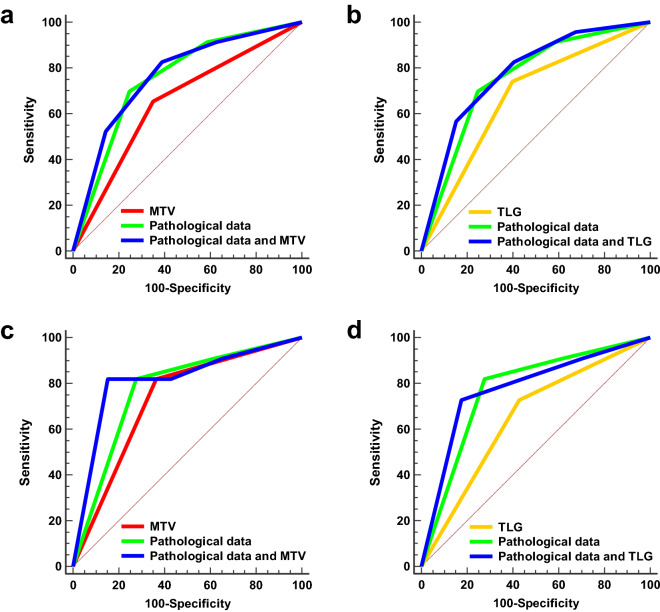
ROC curve analysis of metabolic tumour volume (MTV), total lesion glycolysis (TLG), pathological factor (with pTstage/LN metastasis), model 1 (with pTstage/LN metastasis and MTV), and model 2 (with pTstage/LN metastasis and TLG) for predicting disease-free survival (DFS) and overall survival (OS). Receiving operating characteristic curve analysis showed no additional value of MTV (**a**) and TLG (**b**) to pathologic factor (primary T stage and lymph node metastasis) for predicting DFS. For predicting OS, there was a significant additional value of MTV (**c**), but no additional value of TLG to pathologic factor (**d**).

The prognostic value of each of MTV, TLG, pathological factor (with pTstage/LN metastasis), model 1 (with pTstage/LN metastasis and MTV), and model 2 (with pTstage/LN metastasis and TLG) for the DFS and OS were assessed using the area under the receiver operating characteristic curve (AUC). For DFS, the AUCs of the MTV, TLG, and pathological factor were 0.651 (95% CI 0.569–0.728), 0.671 (95% CI 0.590–0.746), and 0.755 (95% CI 0.678–0.822), respectively. The AUC of model 1 and model 2 using a stepwise multivariate Cox proportional hazard model for DFS were 0.778 (95% confidence interval [CI] 0.702–0.842) and 0.762 (95% CI 0.685–0.828), respectively. There was no significant difference in AUCs for DFS between MTV and pathologic factor (*P* = 0.071), and between TLG and pathologic factor (*P* = 0.173). There was no significant difference in AUCs for DFS between pathologic factor and model 1 (*P* = 0.595), and between pathologic factor and model 2 (*P* = 0.343). For OS, the AUCs of the MTV, TLG, and pathologic factor were 0.728 (95% CI 0.649–0.798), 0.650 (95% CI 0.568–0.726), and 0.773 (95% CI 0.698–0.838), respectively. The AUC of model 1 and model 2 using a stepwise multivariate Cox proportional hazard model for OS were 0.814 (95% CI 0.742–0.873) and 0.779 (95% CI 0.704–0.843). There was no significant difference in AUCs between MTV and pathologic factor (*P* = 0.184), but the AUC of TLG showed a significantly lower value than that of pathologic factor (*P* = 0.030). The AUC of model 1 significantly superior compared to that of pathologic factor (*P* = 0.039) (Fig. 3c), although the AUC of model 2 was not significantly different when compared to that of pathologic factor (*P* = 0.826) (Fig. 3d).

## Discussion

To our knowledge, neither MTV nor TLG had previously been validated as a prognostic factor in patients with CRAC. This study showed the significant prognostic value of preoperative metabolic parameters on ^18^F-FDG PET/CT in patients with CRAC. The MTV and TLG were validated as robust pretreatment prognostic factors for cancer recurrence and cancer-related death. Patients with a high MTV or TLG had a worse clinical outcome than patients with either a low MTV or TLG. Furthermore, the incorporation of MTV with conventional risk factors (high pT stage and positive LN metastasis) showed improved prognostic value compared to conventional risk factors alone. Based on our results, the use of volume-based metabolic parameters in ^18^F-FDG PET/CT could provide more accurate preoperative risk stratification in patients with CRAC.

Volume-based metabolic parameters of ^18^F-FDG PET, including MTV and TLG, have been used for evaluation of prognosis before treatment in patients with various types of cancer. MTV represents volumetric information of the tumour with a relatively high metabolism, while TLG represents a mixed value of the mean SUV and the tumour volume^29^. Thus, MTV and TLG are volume-based ^18^F-FDG PET parameters. The tumour volume could also be provided by CT and MRI, but measurement with these modalities may not be accurate because of the irregular shape of malignant tumours, unclear boundaries, and presence of necrotic component^24^. As MTV and TLG could not only provide volumetric tumour burden but also identify a viable tumour region with metabolic information, these volume-based ^18^F-FDG PET parameters may more accurately reflect the viable tumour volume and grade of malignancy. Furthermore, metabolic tumour delineation is still challenging and numerous PET segmentation methods have been proposed. Among those methods, a fixed absolute threshold of SUV 2.5–5.0 or a fixed relative threshold of 30–60% of the SUVmax were more commonly performed because of simplicity and ease of use, high reproducibility, and observer-independent^29^. A fixed relative threshold of 41% of SUVmax was recommended on the guidelines of European Association of Nuclear Medicine for the case of higher tumour-to-background values and non-heterogeneous ^18^F-FDG uptakes^33^. However, a fixed relative threshold of 50% of SUVmax was recommended when in case of noise, inhomogeneous ^18^F-FDG uptake in the tumour and background, and low tumour-to-background ratios. In the present study, a threshold of 50% of SUVmax for the MTV was chosen because more than half of patients were on advanced T stages that could be causing inhomogeneous ^18^F-FDG uptake, and an area of relatively high ^18^F-FDG uptake (e.g., physiologic bowel uptake, urine radioactivity) was close to/adjacent to the primary tumour.

Previous studies have reported the prognostic role of MTV and TLG in patients with colorectal cancer^21,22,24,26^, but they showed contradictory results on the correlation between the metabolic parameters and cancer prognosis. Deantonio et al.^26^ evaluated the prognostic value of the metabolic parameters on ^18^F-FDG PET for prediction of neoadjuvant chemoradiotherapy response and prognosis in patients with rectal cancer but did not find any correlation with DFS and OS. Another study with ^18^F-FDG PET reported that high MTV and TLG were significantly associated with shorter OS and DFS on univariate analysis, while multivariate Cox proportional hazards analysis revealed that MTV and TLG were not significant potent predictors of DFS and OS^22^. Similar results were reported in patients with locally advanced rectal cancer. Bang et al.^21^ reported that baseline MTV and TLG calculated with various thresholds were significantly associated with 3-year DFS on univariate analysis, but not statistically significant on multivariate analysis. In accordance with our results, Ogawa et al.^24^ reported that the baseline MTV and TLG with an optimal cut-off value calculated by receiver operating characteristic (ROC) curve analysis was significantly associated with 5-year OS on univariate analysis, and high TLG was an independent factor for predicting a poor prognosis on multivariate analysis in patients with colorectal cancer. In the present study, a higher level of MTV and TLG were significantly associated with shorter DFS and OS on univariate analysis, and high MTV was one of the potent predictors of DFS and OS on multivariate analysis. The differences in the results among the previous studies are probably due to several reasons, such as the methods used to determine cut-off values of metabolic parameters, and patient characteristics, such as tumour stages, different follow-up periods, heterogeneous histological types, and various therapeutic modalities after primary treatment. Since colorectal mucinous adenocarcinoma could have a larger primary tumour size than non-mucinous adenocarcinoma^30,31^, volume-based metabolic parameters on ^18^F-FDG PET would be affected by histologic subtypes. In this regard, it is appropriate to separately evaluate prognostic value of the metabolic parameters in the CRAC and other histologic subtypes. In the present study, the volume-based metabolic parameters were evaluated in patients with only CRAC excluding stage IV and this is considered as a cause of this study to have more reliable results than previous studies.

The present study demonstrated that TLG was significantly related to DFS in both univariate and multivariate analyses. TLG showed a significant relationship with OS in univariate analysis, which was not preserved in multivariate analysis. The MTV of the primary tumour was a significant predictive factor for DFS and OS on both univariate and multivariate analyses. A comparison analysis of the AUCs for DFS and OS showed no significant differences between MTV and pathologic factor. Furthermore, a combined model with MTV and pathologic factors showed significantly better accuracy of risk prediction for OS than pathologic factor alone. These results suggest that metabolic parameters of the primary tumour could provide risk stratification in patients with CRAC. The pathologic factor including T and N stages occasionally could not be evaluated before surgery; therefore, ^18^F-FDG PET/CT as a non-invasive imaging modality could provide additional prognostic information in patients with CRAC before surgery.

There were some limitations to the present study. First, this study was a retrospective review, and data were based on a single medical centre with a relatively low number of tumour recurrence, and these issues could have increased the risk of selection bias and influenced the correlation between metabolic parameters and survival. Second, an immunohistochemical investigation of the primary tumour which may be affecting the glucose metabolism of the cancer cell was unavailable. Thus, it was difficult to fully explain the relationship between metabolic parameters and the clinicopathological factors. Therefore, a further multicentre-based prospective study with a large number of patients treated in a homogenous way with a collection of immunohistopathological information of glucose metabolism and observation for a long follow-up time is needed to validate the findings of the current study.

In conclusion, the volume-based metabolic parameters from preoperative ^18^F-FDG PET/CT scans are significant independent prognostic factors in patients with CRAC. The incorporation of MTV with conventional risk factors could provide additional prognostic information in patients with CRAC.

## Methods

### Patients

From February 2009 to June 2016, we enrolled consecutive patients who underwent preoperative ^18^F-FDG PET/CT followed by curative surgeries for CRAC. Patients meeting the following criteria were excluded: patients with other types of malignancies, such as mucinous adenocarcinoma and undifferentiated carcinoma; patients who underwent ^18^F-FDG PET/CT after any treatment including endoscopic mucosal resection, curative surgery, and neoadjuvant chemoradiotherapy; patients with an interval of > 4 weeks between ^18^F-FDG PET/CT and surgery; and patients with distant metastasis. Pathological confirmation of lesions in patients with suspected distant lymph node metastasis was performed if the lesion was anatomically accessible by biopsy. However, the suspected metastatic lymph node in the retroperitoneum or other region, which is anatomically difficult to access, was clinically determined by careful discussion at a regular multidisciplinary conference for patients with colorectal cancer. Clinicopathologic data considered to be potentially important to prognosis were collected from the patients’ electronic medical records. The clinicopathologic data included age at surgery, sex, CEA level before surgery, pT stage, pathologic N (pN) stage, LVI, PNI, and use adjuvant chemotherapy after surgery. The classifications of pT and pN were conducted according to the seventh edition of the American Joint Committee on Cancer/International Union Against Cancer. The pT stage was divided into low (T1 and T2) and high (T3 and T4) T stage groups, and the pN stage into negative (pN0) and positive (pN1 and pN2) groups according to the presence of LN metastasis. The Institutional Review Board of Keimyung University Dongsan Hospital approved the current study (approval number: 2018-12-024). A written informed patient consent was waived by the Keimyung University Dongsan Hospital Institutional Review Board due to the retrospective nature of study.

### Treatment protocol

The indication for neoadjuvant chemoradiotherapy at our institution included a histologically proven adenocarcinoma of either the mid or distal rectum that was clinically staged as T3/T4 or LN-positive after preoperative work-up. Neoadjuvant chemoradiotherapy was administered using 5-fluorouracil and leucovorin with concurrent radiation, and the total radiation dosage was 5,040 cGy in 25 fractions delivered over 5 weeks. The operation was to be performed within 6–8 weeks after completion of neoadjuvant chemoradiotherapy. Surgical treatment was performed based on the principles of total mesorectal excision. The protocol for adjuvant chemotherapy of patients followed the National Comprehensive Cancer Network guidelines^32^.

### ^18^F-FDG PET/CT scan

^18^F-FDG PET/CT scan was performed using either the Discovery STE-16 (GE Healthcare, Milwaukee, WI, USA) or the Biograph mCT-64 (Siemens Healthcare, Knoxville, TN, USA) scanner. The patients were asked to fast for at least 6 h before the scan, and serum glucose levels in the peripheral blood were checked until they were lower than 150 mg/dL before ^18^F-FDG was injected. All patients with diabetes were asked to discontinue their antihyperglycemic drugs 12 h before the scan. Patients received 4.0 MBq (0.11 mCi)/kg (Biograph mCT-64) and 7.0 MBq (0.19 mCi)/kg (Discovery STE-16) of ^18^F-FDG intravenously according to the PET/CT system. After 1 h of ^18^F-FDG uptake, an initial low dose non-contrast CT scan was obtained for attenuation correction and localization. Immediately after the CT scan, standard PET images were acquired from the base of the skull or top of the brain to the proximal thigh in three-dimensional mode. The Discovery STE-16 PET/CT scanner acquired images with a slice thickness of 3.75 mm simultaneously for a longitudinal field of view (FOV) of 780 mm. The transaxial FOV was 700 mm, and the matrix size was 128 × 128. Spatial resolution in the air was 4.29 mm full-width half-maximum (FWHM). The PET images were reconstructed from CT data for attenuation correction using the ordered subset expectation–maximization (OSEM) iterative algorithm with 20 subsets and two iterations. Meanwhile, the Biograph mCT-64 PET/CT scanner acquired images with a slice thickness of 3 mm simultaneously for a longitudinal FOV of 500 mm. The transaxial FOV was 58.8 cm, and the matrix size was 256 × 256. Spatial resolution in the air was 4 mm FWHM. The PET images were reconstructed from CT data for attenuation correction using the TrueX algorithm and an all-pass filter with 21 subsets and two iterations.

**Figure 4:**
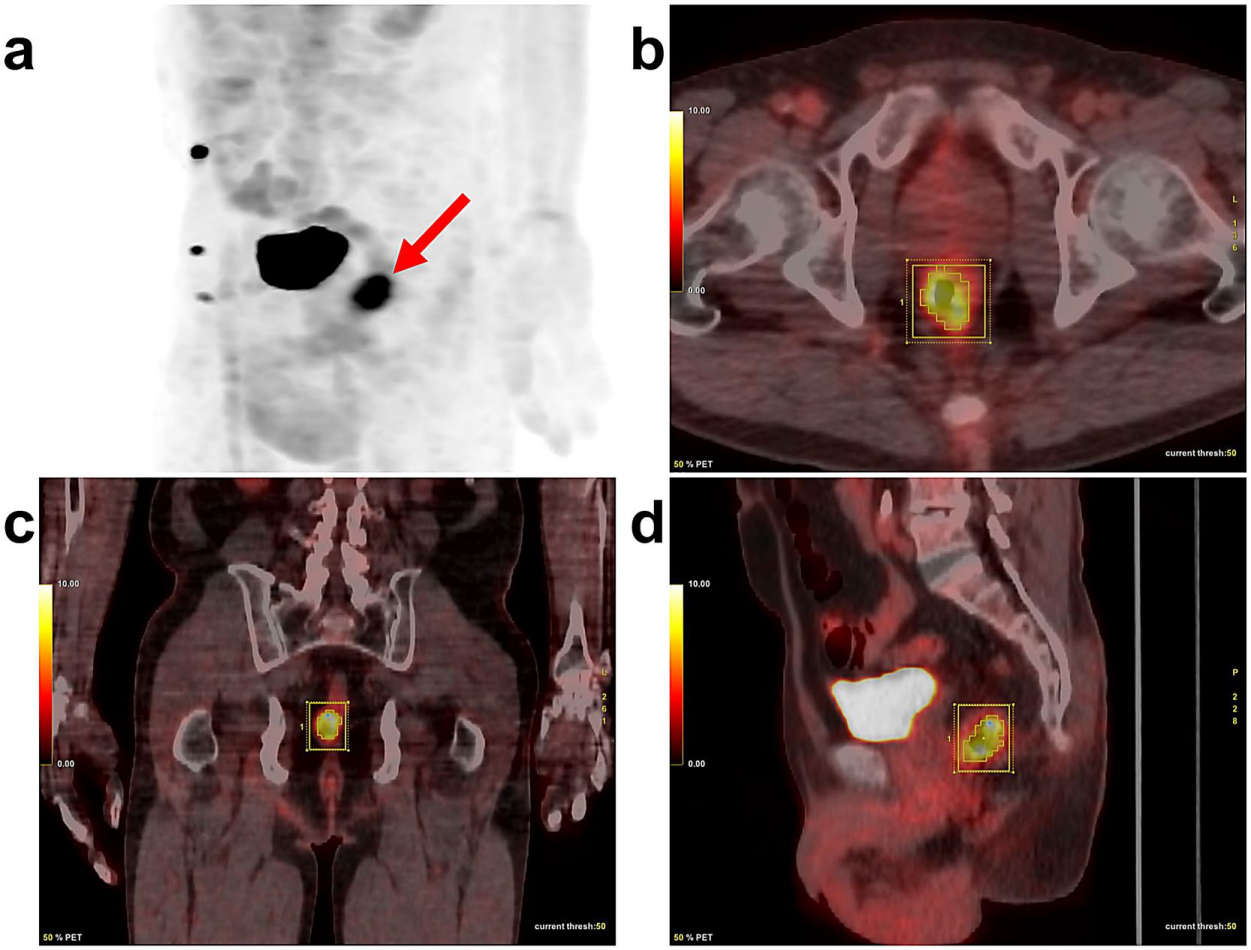
Representative case for the measurement of the maximum standardized uptake value (SUVmax), metabolic tumour volume (MTV), and total lesion glycolysis (TLG) in a 64-year-old male patient with rectal cancer. The maximum intensity projection image shows well-demarcated primary rectal cancer lesion (**a**) (arrow). We drew a volume of interest to sufficiently surround the primary rectal cancer lesion on transaxial (**b**), coronal (**c**), and sagittal (**d**) images. Then the software automatically calculated the SUVmax (10.40), MTV (7.97 cm^3^), and TLG (56.3 g).

All metabolic parameters, including the SUVmax, SUVmean, MTV, and TLG, were automatically obtained using each dedicated PET workstation (ADW version 4.3 for Discovery STE-16). The CT images were taken by excluding physiologic uptake of adjacent organs and vessels by considering the tumour location. The SUVmax and SUVmean were separately measured as the highest and mean values of SUV in all discernible primary lesions of each patient by drawing a volume of interest on the PET images (Fig. 4). The MTV of the primary lesion was defined as the total tumour volume greater than 50% of the SUVmax^33^. TLG was calculated by multiplying the MTV and the SUVmean of the MTV. The SUVmax, MTV, and TLG were dichotomized using the optimal cut-off values by ROC curve analysis. If the ROC curve analysis did not show any statistical significance in the optimal cut-off values for prediction of DFS and OS, the metabolic parameter was dichotomized at its median value. The patients were divided into two groups according to the optimal cut-off values of the metabolic parameters.

### Follow-up

During the follow-up period, physical examination, laboratory tests for tumour markers, and a CT scan of the chest and abdominopelvic cavity were performed as part of the routine follow-up protocol. DFS was defined as the interval from surgery to tumour recurrence or final medical examination for recurrence evaluation if recurrence did not occur. When recurrence of the disease was suspected, ^18^F-FDG PET/CT and chest and abdominopelvic cavity CT were used for the diagnosis of the disease. OS was defined as the time from the start of the main treatment to either death or the last follow-up visit at our institution. Survival status was retrieved from electronic medical records or attempts to contact the referring physicians.

### Statistical analysis

All statistical analyses were performed using the IBM Statistical Package for the Social Sciences for Windows version 26.0 (IBM Corp., Armonk, NY, USA) and MedCalc Statistical Software version 19.4.0 (MedCalc Software Ltd, Ostend, Belgium; https://www.medcalc.org; 2020). A Kaplan–Meier analysis with a log-rank test was performed for univariate survival analysis. Significant variables (*P* < 0.05) from the univariate analysis were entered into a multivariate analysis that was performed using a Cox proportional hazard model. A Spearman rank correlation coefficient was obtained before the multivariate analysis for the evaluation of the multicollinearity between MTV and TLG. The comparison between predictive models was evaluated using the AUC. The additional value of the MTV for prognostication was also evaluated using the AUC. The Shapiro–Wilk test was used for the evaluation of normality of data. A *P* value of < 0.05 was considered to be statistically significant.

## Data Availability

The datasets generated during and/or analysed during the current study are available from the corresponding author on reasonable request.
